# Which positive personality traits do people want to change?

**DOI:** 10.1177/08902070231211957

**Published:** 2023-11-11

**Authors:** Fabian Gander, Lisa Wagner

**Affiliations:** 1 Department of Psychology, 27209 University of Basel, Basel, Switzerland; 2 Department of Psychology, 27217 University of Zurich, Zurich, Switzerland; 3 Jacobs Center for Productive Youth Development, 27217 University of Zurich, Zurich, Switzerland

**Keywords:** personality change, volitional trait change, character strengths, change goals, well-being

## Abstract

Most people want to change some of their personality traits, typically those they and others perceive as lacking. However, past research focused on student samples and higher-order traits and has not fully explored the attributes of traits that predict change goals. As a replication and extension of previous findings, two studies examined (1) whether people want to change their character strengths and how character strengths change goals relate to (2) character strength levels, (3) age, and (4) well-being. Further, we examined which (5) attributes of character strengths, such as their association with morality or well-being, predict change goals. Participants (Study 1: *N* = 2,792 German-speaking adults, 79.2% women, median = 46 years; Study 2: *N =* 6,787 English-speaking adults, 67.0% women, median = 32 years) completed measures of character strengths, character strengths change goals, and well-being. A subsample (*n* = 1,739) provided informant ratings. Results showed that participants wanted to increase all 24 character strengths. Most change goals showed negligible associations with participants’ well-being and age. Except for spirituality, participants—especially the less happy—wanted to change those character strengths they lacked. The character strengths’ relationship with well-being, but not their moral value, predicted the goals to change them.

## Introduction

Accumulating evidence suggests that most people want to change at least some of their personality traits (e.g., Hudson & Roberts, 2014; Miller et al., 2019). Across many countries, an average of 60% of young adults currently want to change their personality (Baranski et al., 2021). Such goals to change personality traits robustly predict longitudinal changes in personality (Hudson et al., 2020). Most studies have focused on student samples and examined goals to change relatively few higher-order traits, such as the Big Five or HEXACO personality traits. In addition, little is known about what attributes of traits might drive the goals to change them. In the present set of two studies, we aim to replicate the knowledge on goals to change personality by using two online samples of adults, one of which consisted of people with the intention to increase their well-being. We examine the associations of change goals to individual differences such as trait levels, age, and well-being. Further, we aim to extend previous knowledge by examining whether attributes of traits, such as their normativity or their associations with morality and well-being, predict change goals. For this purpose, we are studying goals to change a set of 24 narrower, positively valued traits, namely, character strengths, as described by the VIA classification (Peterson & Seligman, 2004).

The VIA classification is a product of a collaborative effort to describe positively valued traits that contribute to the “good life” for oneself and others (Peterson & Seligman, 2004). This list of 24 character strengths has been used in numerous studies. Character strengths are valued across cultures (e.g., Pievsky & McGrath, 2022) and robustly associated with well-being (e.g., Bruna et al., 2019; Wagner et al., 2020). They are overlapping, but not redundant with, Big Five and HEXACO personality domains and facets (McGrath et al., 2020; Ruch et al., 2023). Character strengths are considered trait-like yet malleable (Peterson & Seligman, 2004). Even though little research has considered changes in character strengths (Gander et al., 2020; Gander & Wagner, 2022), character-based strength interventions are designed to change people’s character strengths (e.g., Ruch et al., 2020). Such interventions typically do not allow participants to select which character strengths they want to change but either (a) target specific strengths, for instance, gratitude, kindness, or humor, or more generally, those character strengths with the strongest relationships to well-being (“generalized interventions”; e.g., Proyer et al., 2013), or (b) focus on the individual’s highest or lowest strengths (“personalized interventions”; e.g., Proyer et al., 2015). But do people even want to change in these ways? And which character strengths are they particularly interested in changing? Therefore, studying which character strengths people want to change can not only inform research on volitional personality change but also applied interventions.

### Who wants to change which traits?

People tend to want to change the personality traits they (relatively) lack. This finding has been replicated using different methodological approaches for assessing change goals (e.g., Costantini et al., 2020; Hudson & Fraley, 2016a; Hudson & Roberts, 2014; Miller et al., 2019; Robinson et al., 2015) and both for self- and informant ratings on trait levels (Quintus et al., 2017; Sun & Goodwin, 2020). However, the relationship between trait levels and change goals differs between traits. Summarizing previous studies on the relationships between change goals and trait levels for the Big Five personality traits, Thielmann and de Vries (2021) concluded that studies typically find strong negative correlations for extraversion, neuroticism, and conscientiousness but only a weak negative correlation for agreeableness and a zero correlation for openness. Going beyond the Big Five personality traits, honesty/humility, as conceptualized in the HEXACO framework, showed a zero correlation between trait levels and change goals (Thielmann & de Vries, 2021). In addition, the Dark Triad traits showed either a zero (Psychopathy and Narcissism) or even a positive (Machiavellianism) correlation between trait levels and change goals (Hudson, 2022).

Similarly, in a study explicitly addressing a range of morally relevant traits, Sun and Goodwin (2020) found that the negative relationship between traits and change goals was weaker for traits with higher moral relevance than for traits with lower moral relevance. These findings suggest that goals to change morally valued traits are mostly unrelated to people’s levels of these traits. Another suggestion was put forward by Thielmann and de Vries (2021), who argued that differences among traits could partially be explained by a trait’s (perceived) social desirability.

In this study, we examine individuals’ goals to change positively valued traits using the VIA classification of character strengths (Peterson & Seligman, 2004), a comprehensive framework of narrower, positively valued traits that include morally relevant traits. Studying goals to change character strengths allows for replicating and extending previous findings on personality change goals. In particular, our study can provide insights into the recently discussed questions of which traits have a negative relationship with goals to change them (e.g., Sun & Goodwin, 2020; Thielmann & de Vries, 2021). By using a comprehensive framework of narrower, positively valued traits, our study can increase the generalizability of findings on change goals.

### Are goals to change personality related to age?

Emerging and young adulthood has been associated with the most substantial changes in personality traits (Bleidorn et al., 2022; Roberts et al., 2006). Perhaps as a consequence but also out of convenience, most research on personality change goals has focused on young adults, typically college students or individuals younger than 30. Indeed, when comparing change goals between a sample of young adults (on average 21 years old) and a sample of older adults (on average 68 years old), Quintus et al. (2017) found that overall, the younger sample reported stronger goals to change their personality traits than the older sample. This difference was found for all Big Five personality traits but was most pronounced for conscientiousness. This result is also in line with earlier findings showing that (a) older adults generally had less pronounced personality change goals and (b) the largest age-related differences in change goals were observed for conscientiousness, emotional stability, and extraversion while goals to change agreeableness and openness showed little age-related differences (Hudson & Fraley, 2016b). As a potential driver of age-related differences in change goals, developmental tasks that vary across the life span (Hutteman et al., 2014) have been put forward. However, regardless of these age-related differences, goals to change personality traits are also pervasive in middle and older adulthood (Hudson & Fraley, 2016b; Quintus et al., 2017). Thus, it seems relevant to study goals to change personality traits across the whole lifespan instead of only in college students.

For the character strengths of the VIA classification, a meta-analysis on cross-sectional age differences found higher scores for older people in most character strengths (Heintz & Ruch, 2022). In addition, differential relationships between character strengths and well-being have been found across different ages (Baumann et al., 2020; Martínez-Martí & Ruch, 2014). These findings indicate that the relevance of character strengths for well-being might vary as a function of age or challenges faced in the current life stage. In the present study, we investigated how character strengths change goals related to age, enabling us to investigate the variations of character strengths change goals across the lifespan. Age-related differences in character strengths people want to change would be in line with the idea that the relevance of certain character strengths might change as we get older and are faced with different developmental tasks (Hutteman et al., 2014).

### Do personality change goals relate to well-being?

Why do people want to change their personality traits? One frequently voiced assumption is that wanting to improve one’s well-being can be a driving force for desires to change one’s traits (e.g., Sun & Goodwin, 2020). Indeed, individuals with lower well-being tend to report stronger goals to change personality traits (Hudson & Fraley, 2016a). However, when controlling for trait levels (which are also associated with well-being; see Anglim et al., 2020, for a recent meta-analysis), only goals to increase conscientiousness robustly showed negative associations with well-being (Hudson & Roberts, 2014; Quintus et al., 2017). That means people who were less satisfied with their lives tended to report stronger goals to increase conscientiousness (but not other traits that are also related to well-being).

Almost without exception, previous studies on goals to change personality traits have conceptualized well-being as life satisfaction, the cognitive evaluation of one’s life circumstances. We used a comprehensive measure of well-being (Su et al., 2014), which encompasses both dimensions of subjective well-being (life satisfaction, and positive and negative affect) and eudaimonic well-being (positive relationships, interest and engagement in daily activities, meaning and purpose in life, a sense of mastery and accomplishment, feelings of control and autonomy, and optimism). Hudson and Roberts (2014) found that dissatisfaction with a particular life domain was associated with goals to change personality traits associated with that life domain (e.g., being dissatisfied with friendships was linked with goals to become more extraverted, agreeable, and emotionally stable, but not with goals to become more conscientious or open, whereas being dissatisfied with school was most strongly linked with goals to become more conscientious). It is thus conceivable that well-being dimensions also relate differentially to change goals. Concretely, in Study 1, we investigated whether people low on certain dimensions of well-being would report goals to change character strengths specifically related to these dimensions (e.g., Hausler et al., 2017b; Wagner et al., 2020).

Most studies on goals to change personality traits used samples of young adults who participated for course credit or received payment. One notable exception is the study by Stieger et al. (2020), which examined change goals in an intervention study aimed at changing the Big Five traits. Similarly, Study 1 used a sample of adults from the general population who signed up for an online intervention advertised as aimed at increasing well-being. For about a third of the sample in Study 1 (i.e., those who were assigned to the intervention condition), their stated change goals had direct consequences because the subsequent training program was based on the character strengths for which participants indicated the strongest desire to change. We, therefore, expect the results to more validly reflect participants’ true preferences as compared to a sample who did not explicitly express interest in any changes. Study 2 was designed to test the robustness of the findings in people who had not actively enrolled in an intervention study and included adults from a broad age range.

Goals to change personality traits are relevant to well-being beyond the effect of the personality traits themselves (Hudson & Roberts, 2014). But does the interaction between traits and change goals also relate to well-being? For instance, it is conceivable that a strong desire to change a trait is more strongly related to the individual’s level of the respective trait if the individual scores low on well-being compared to when they score high. Research on the association between personality traits and goals (Reisz et al., 2013) suggests that traits and goals can be related to each other in a *compensatory* (e.g., a person low in self-regulation wants to get better at exercising regularly) or a *complementary* (e.g., a person high in love of learning wants to participate in a continuous education course) fashion. When applying this idea to goals to change personality traits, a compensatory goal would represent the desire to increase a trait in which a person is low, and a complementary goal would represent the desire to increase a trait in which a person is already high. In the present study, we will investigate whether well-being predicts whether the relationship between change goals and traits is rather compensatory or rather complementary; that is, whether the relationship between character strengths traits and change goals depends on well-being.

### Do attributes of traits contribute to change goals?

Sun and Goodwin (2020) proposed that people are less inclined to change moral traits. Since character strengths represent a broad range of desirable traits that differ in their moral value (e.g., kindness as a highly morally valued trait vs. creativity as a desirable but less morally valued trait; see Stahlmann & Ruch, 2020), they lend themselves well to examine this notion further. We used existing data on correlations between self-ratings of character strengths and moral character (Wagner et al., 2021) for an estimation of the moral value of each character strength as a predictor of change goals.

According to Hudson and Fraley (2016b), individuals who are unhappy with certain aspects of their lives may set goals to improve traits they believe will alleviate their dissatisfaction. In our study, we aimed to investigate whether individuals are more interested in changing character strengths that are strongly associated with well-being. Additionally, we included normative levels of character strengths (Ruch et al., 2010) to determine whether people want to become more similar to the average level of character strengths and the national valuing of each character strength (Pievsky & McGrath, 2022) to determine whether individuals seek to change in ways that are culturally valued, which is related to the desirability of a trait (Thielmann & de Vries, 2021). By exploring whether the moral value of a trait contributes to individuals’ desire to change it and contrast this idea with other possibilities, such as whether change goals are related to a trait’s expected association with well-being or its normative or nationally desirable levels, our study contributes to the ongoing discussion on which attributes of traits drive the desire to change them (Sun & Goodwin, 2020; Thielmann & de Vries, 2021).

### Overview and aims of the present studies

In the present set of two studies, we investigate goals to change positively valued personality traits using the VIA classification of character strengths (Peterson & Seligman, 2004) as a theoretical framework. In our two studies, we examined five main research questions: We investigated (1) the prevalence of goals to change character strengths, and how character strengths change goals related to (2) levels of character strengths, (3) age, and (4) well-being, and (5) we examined which attributes of traits might predict change goals. For ease of reading, we present Study 1 and Study 2 together.

#### Aims of Study 1

In Study 1, we investigated research question 1 by analyzing the ratings of change goals. Additionally, we examined—for a subsample who participated in a strengths-based intervention program—the character strength that was selected for the training.^1^ This was meant to serve as a behavioral indicator for the goal to change a character strength. Research question 2 was examined by studying the associations between self- and informant-rated character strengths with change goals. In addition to linear associations, we also examined quadratic associations of character strengths traits and the relationships of traits to absolute levels of change goals (i.e., the average change goals across all strengths, regardless of direction). Research question 3 was examined by studying the associations between change goals and age. For research question 4, we examined the associations of change goals with a multidimensional framework of well-being. Additionally, we tested whether the relationship between character strengths levels and character strengths change goals depends on well-being. Finally, for research question 5, we examined whether attributes of character strengths, namely, character strengths’ association with moral value and well-being, their normative levels, and the extent to which they are valued are predictive of change goals, above the influence of character strengths trait levels and well-being.

Since Study 1 was the first to address change goals related to character strengths, it was of a descriptive and exploratory nature. Therefore, we preregistered the research questions (except for research question 5) and the analysis plan but did not formulate specific hypotheses (https://osf.io/cew4r).

#### Aims of Study 2

The main purpose of Study 2 was to replicate the main findings of Study 1. We preregistered our hypotheses and the analysis plan for Study 2 (https://osf.io/tu8fw).

In Study 2, we again tested research question 1 by examining the ratings of change goals. We expected that, on average, people wanted to increase all character strengths. For research question 2, we again examined correlations between self-rated character strength levels and change goals. Based on the findings of Study 1, we expected negative relationships for all strengths but a positive relationship for spirituality. For research question 3, we examined the partial correlations between change goals and age, adjusted for trait levels. We expected negative relationships for most strengths, in particular for leadership, gratitude, perseverance, and zest. For research question 4, we examined the partial correlations between change goals and well-being, adjusted for trait levels of character strengths. We expected negative relationships for most strengths, in particular for hope, spirituality, appreciation of beauty and excellence, zest, bravery, and love of learning. Finally, for research question 5, we examined, as in Study 1, whether attributes of character strengths (i.e., character strengths’ associations with moral value and well-being, their normative levels, and the extent to which they are valued) are predictive of change goals. In addition to Study 1, we also considered participants’ subjective expectations regarding each strength’s contribution to their well-being. We expected that both a trait’s association with well-being and a trait’s subjective contribution to well-being would positively relate to the magnitude of change goals.

## Methods

For both studies, we report how we determined our sample size, all data exclusions, all manipulations, and all measures in the study. We preregistered an analysis plan for Study 1 before accessing the data (https://osf.io/cew4r) and preregistered the hypotheses and the analysis plan for Study 2 before data collection (https://osf.io/tu8fw). The data for Study 1, the code underlying all analyses, supplementary Tables and Figures, and a document detailing the deviations from the preregistrations for Studies 1 and 2 are available from the project’s OSF page (https://osf.io/2xfyq/). Due to the data privacy policy of the institute that collected the data for Study 2, the data, unfortunately, cannot be shared.

### Study 1

#### Participants

As stated in the preregistration, we did not determine the sample size in advance but used all data available on January 22, 2022. Sensitivity analyses (α = .05, power = .80, two-tailed tests) suggested that the minimal detectable effect sizes were *d* = .05 for the analysis testing whether people do want to change their strengths (one-sample *t*-test; corresponding to *r* = .03). For the analysis examining correlations with traits, age, and well-being, the minimal detectable effect sizes were *r* = .05 (self-ratings) and *r* = .07 (informant ratings). We did not conduct sensitivity analyses for the multilevel models.

The sample consisted of German-speaking adult participants who were currently undergoing neither psychological nor psychopharmacological treatment and were currently not depressed (i.e., scores below 15 in the PHQ-9, Kroenke et al., 2001). These preregistered screening criteria were implemented in the survey because the data presented here was collected as part of the baseline assessment of an online intervention study. Individuals who did not meet these criteria did not advance in the survey (and 11 participants were additionally removed because they did not meet the criterion of being at least 18 years old). In total, 2,823 participants and 1,932 informant raters provided data on all study variables. As preregistered, we excluded data from 31 participants and 40 informant raters who completed the survey unusually fast (i.e., two times faster than the median completion time; Leiner, 2019), and 153 informant raters could not be matched to the participants.

Thus, the samples consisted of *N* = 2,792 participants (20.5% men, 79.2% women, 0.4% indicated “other”) and *N* = 1,739 informant raters (48.6% men, 51.1% women, 0.3% indicated “other”). The age distribution of both samples was very similar (participants: *Md* = 46 years, *SD* = 13.74, range: 18–90; informant raters: *Md* = 46 years, *SD* = 14.48, range: 18–84). Around two-thirds (62.4%) of the participants reported holding a degree from either a university or a university of applied sciences. Only a small proportion (13.9%) of the participants indicated currently being enrolled in part-time or full-time studies.

Informant raters were mostly the participants’ partners (55.7%), friends (22.7%), parents (7.8%), children (5.3%), or siblings (4.6%). They knew the participants on average for 20.7 years (*SD* = 13.77) and indicated that their relationship was “very close” on average (*M* = 6.08 on a 7-point Likert scale ranging from 1 = “not at all close” to 7 = “extremely close”).

#### Procedure

Data for this study were collected within a larger intervention study. The intervention study included three conditions: character strengths training, placebo control, and waitlist control. The present study uses data collected at the baseline assessment. Since participants were assigned to the conditions only at the end of this baseline assessment (i.e., after completing the measures of character strengths, well-being, and character strengths change goals), the assignment did not influence the present results. Participants were recruited from the community through social media ads, news articles, flyers, and mailing lists. They signed up for the intervention study, provided informed consent, and were then asked to complete the baseline assessment. This assessment contained all measures reported here and additional measures not relevant to the current study (namely, a depression screening measure, a measure of personality, questionnaires on character strengths-related behavior in the last week, their experience of positive emotions in general and in the last week, and the participants in the training condition additionally selected a character strength that they wanted to focus on in the training and rated their subjective beliefs about the malleability of the character strength they had selected). After having completed the baseline assessment, participants were asked to send the link to the informant rating to a close other to obtain informant ratings. Participants and informant raters provided informed consent, and participation was voluntary. The study protocol was approved by the ethics committee of the Faculty of Arts and Social Sciences of the University of Zurich (approval no. 19.12.22).

### Instruments

#### Character strengths levels

To assess self-rated levels of character strengths, we used the *Values in Action Inventory of Strengths* (VIA-IS; Peterson & Seligman, 2004; German version by Ruch et al., 2010). It consists of 240 items (10 items per strength) using a 5-point Likert-style scale (from 1 = “very much unlike me” to 5 = “very much like me”). In the present study, internal consistencies ranged between ω = .73 (kindness) and ω = .91 (spirituality).

To assess informant-rated levels of character strengths, we used a shortened version of the VIA-IS (i.e., the VIA-IS-120; Höfer et al., 2020). It consists of 120 items (5 items per strength) adapted to be used as informant ratings for the present study. Höfer et al. (2020) showed that this shortened version converges well with the original 240-item VIA-IS. In the present study, internal consistencies ranged between ω = .67 (perspective) and ω = .87 (spirituality). Informant ratings converged well with self-ratings; the median correlation across all character strengths was *r* = .37 and ranged from *r* = .17 (honesty) to *r* = .61 (spirituality). Further, all convergent correlations between self- and informant ratings were higher than discriminant correlations, except for informant ratings of honesty, which correlated slightly higher (*r* = .18) with self-ratings of modesty. Thus, the convergence is very similar to the self-informant convergence reported by Ruch et al. (2010) using the mean across two informants and a 240-item informant report (median self-informant correlation: *r* = .41).

#### Well-being

To assess well-being, we used the Comprehensive Inventory of Thriving (CIT; Su et al., 2014; adapted to German by Hausler et al., 2017). The CIT covers both subjective and psychological well-being and consists of 54 items (18 subscales and seven higher-order scales). In the present study, we used six higher-order scales (relationships, engagement, mastery, autonomy, meaning, and optimism), and the three subscales summarized into subjective well-being (life satisfaction, positive feelings, negative feelings). We adapted the answer format from a 5-point to a 7-point Likert-style scale (from “1” = strongly disagree to “7” = strongly agree) to fit with other questionnaires used in this part of the survey. Internal consistencies ranged between ω = .77 (engagement) and ω = .96 (mean score across all scales).

#### Change goals

To assess character strengths change goals, we asked participants to indicate the extent to which they want to change each of the 24 character strengths. This measure (Character Strengths Change Goals Rating Form) is based on the Character Strengths State Rating Form (Gander et al., 2022) and uses one item per strength containing a description of the respective trait. For this study, we changed the response scale to a 5-point scale (ranging from −2 = “much less than I currently am” through 0 = “no change” to 2 = “much more than I currently am”).

After having indicated their character strengths change goals, those who were randomly assigned to the character strengths training condition (*n* = 1,018) could select one character strength that they wanted to focus on in the training program, that is, that they actively wanted to increase in the upcoming eight weeks. These participants were presented with a list of five character strengths to make their selection. This list consisted of the five character strengths for which the participant had indicated the strongest change goals (in the direction of increase) in the Character Strengths Change Goals Rating Form. Possible ties were resolved by a random selection among the highest-rated character strengths.

#### Attributes of character strengths

Traits can vary regarding numerous attributes, for example, regarding their desirability, their frequency, or their relationships with outcomes. For example, kindness is on average considered more desirable than spirituality. In addition to characteristics of individuals, we also studied the role of attributes of character strengths in explaining variation in character strengths change goals. In Study 1, we tested whether goals to change character strengths depended on how strongly a character strength was morally valued (*moral value*), how strongly it correlated with well-being (*association with well-being*), how it is distributed in the population (*normative level*) and how strongly it is valued in society (*valuing*).

We obtained estimates of the *moral value* of each character strength from unpublished data associated with Wagner et al. (2021). In their study, Wagner and colleagues (2021) assessed character strengths levels using the VIA-IS and a measure for the self-assessment of moral character: Participants rated the item (abbreviated) “Ethical virtues or character virtues: This refers to the extent to which people act in a morally correct and responsible manner and always have the good of others in mind. Ethical virtues make it possible to direct one’s own behavior toward the good. How would you rate your own expression in this area?” on a 9-point scale, ranging from 1 = “Ethical virtues very low” to 9 = “Ethical virtues absolutely excellent/outstanding.” To obtain a score of the moral value of each character strength, we computed the correlations between this rating and the VIA-IS scale scores and transformed the correlations to Fisher’s Z scores. That is, this estimate describes how strongly a character strength is related to a global rating of moral character and allows testing whether the desire to change a character strength is related to how closely it is related to moral character.

Estimates of the *associations with well-being* of each character strength were obtained by computing the (Fisher’s Z transformed) correlations of the character strengths levels with the well-being measure (i.e., the CIT) in the present study. That is, this estimate describes how strongly a character strength is related to well-being and allows testing whether the desire to change a character strength depends on its association with well-being.

For estimates of the *normative levels* of character strengths, we used the mean levels of character strengths reported by Ruch et al. (2010) for the German version of the VIA-IS (as used in the present study) in a sample similar in demographic composition to the present study. That is, this estimate describes how the character strengths are typically distributed and allows testing whether the desire to change a character strength is related to wanting to become more similar to the typical level of a character strength.

For estimates of the *valuing* of character strengths in society, we used the mean levels from the ratings from Western Europe presented by Pievsky and McGrath (2022). Pievsky and McGrath (2022) asked participants to rate the extent to which they thought each of the 24 strengths was valued in their country on a 5-point scale from 1 = “not at all” to 5 = “very well.” That is, this estimate describes how strongly a character strength is seen as valued in Western Europe and allows testing whether the desire to change a character strength is related to how strongly it is perceived as valued in society.

### Study 2

#### Participants

We aimed at recruiting at least 620 participants since this sample size would have allowed us to detect small effects (*r* ≥ .1) with a power of > .80. Exclusion criteria were being below 18 years old and failing an instructed response attention check (“for this item, please mark ‘more than I currently am’”).

A total of *N* = 6,787 participants fulfilled the criteria. Since providing demographic information was optional, only two-thirds of the sample answered most of the demographic questions. Of these participants, most identified as women (67%), 30% as men, 2% as non-binary, and 1% preferred not to say. Participants were aged 18–86 years (*Md* = 32, *SD* = 13.82). Participants were predominantly from the United States (56%), Australia (10%), the United Kingdom, the Philippines, or Canada (each 6%), while the remaining 16% were from all over the world. About a third of the sample had a Bachelor’s degree (31%), 24% had a Master’s degree, doctorate, or professional degree, 17% had some college but no degree, 10% had a high school degree, and the remaining 18% had a different education (i.e., graduate/professional school, associate’s degree, certificate/technical degree, or less than high school degree).

#### Procedure

According to the guidelines of the local ethics committee, the present study was exempt from ethics review. The data was collected online between January 31 and February 5, 2023, by the VIA Institute on Character. On their website, participants can complete character strengths surveys free of charge and receive automated feedback on their character strengths. After the character strengths survey, participants were invited to complete additional questions for the purpose of this study before receiving feedback on their character strengths. All participants provided written informed consent. They did not receive any remuneration for their participation.

### Instruments

#### Character strengths levels

To assess self-rated levels of character strengths, we used the VIA-IS-P (McGrath, 2019), which contains 96 positively keyed items for the assessment of the 24 character strengths of the VIA classification with four items per strength. It uses a 5-point scale ranging from 1 = “very much unlike me” to 5 = “very much like me.” We only received average scores of each scale and, therefore, could not compute internal consistency in the present study. However, previous research conducted in very similar settings as the present study has demonstrated sufficient internal consistency. A recent review (McGrath, 2023) reported a median of ω = .79 for the VIA-IS-P.

#### Well-being

To assess well-being, we used the Brief Inventory of Thriving (BIT; Su et al., 2014), which is the short measure of the scale used in Study 1. The BIT covers subjective and psychological well-being and has ten items that are answered on a 5-point scale ranging from 1 = “strongly disagree” to 5 = “strongly agree.” Internal consistency was ω = .92.

#### Change goals

To assess character strengths change goals, we asked participants to indicate the extent to which they want to change each of the 24 character strengths. This measure is based on the Signature Strengths Survey (McGrath, 2019) and uses one item per strength containing a description of the respective trait. The measure is highly similar to the one used in Study 1 and already existed in English. For this study, we used a 5-point response scale ranging from −2 = “much less than I currently am” through 0 = “no change” to 2 = “much more than I currently am."

#### Attributes of character strengths

Estimates of the *associations with morality* of each character strength were identical to Study 1. Estimates of the *association with well-being* of each character strength were obtained by computing the (Fisher’s Z transformed) correlations of the character strengths levels with the well-being measure in Study 2 (i.e., the BIT). For estimates of the *normative levels* of character strengths, we used the mean levels reported by McGrath and Wallace (2021) for the English version of the VIA-IS-P, which was used in this study. For estimates of the *valuing* of character strengths, we used the mean levels from the ratings from North America presented by Pievsky and McGrath (2022).

To assess the *subjective contribution of character strengths to well-being*, we asked participants to rate the extent to which each of the character strengths is good or bad for a person’s happiness and well-being. We used the same descriptions of character strengths as for the change goals. The measure used a 7-point scale ranging from 1 = “very bad for own well-being” through 4 = “neither good nor bad” to 7 = “very good for own well-being.” That is, this estimate describes how strongly the study participants perceived a character strength to contribute to one’s well-being and allows testing whether the desire to change a character strengths depends on one’s belief that it contributes to well-being.

Descriptive statistics for character strengths, well-being, and the subjective contribution of character strengths to well-being are provided in Table S1B.

### Data analysis

As preregistered, we used the guidelines by Gignac and Szodorai (2016) to interpret effect sizes and only interpret effects that meet the threshold for a small effect, that is, that are at least *r* ≥ .10 (the smallest effect size of interest in the current study, corresponds to |*d*| ≥ .20), while *r* ≥ .20 (corresponds to |*d*| ≥ .41) denotes a medium, and *r* ≥ .30 (corresponds to |*d*| ≥ .63) a large effect. Deviating from the preregistration of Study 1, we do not report *p*-values but instead interpret confidence intervals for all parameters.^2^

For examining research question 4 (examining whether the relationships between character strengths levels and change goals depend on well-being), we conducted multilevel models. Therefore, we transformed the data to the “long” format so that the data set contained 24 rows for each person (one row for each character strength, with separate columns for traits, change goals, and well-being, respectively). We separated character strengths trait levels into parts that vary between person (i.e., a person’s average level across all character strengths) and the parts that vary within persons (i.e., a person’s deviation in each strength from their average level across all character strengths). Within-person parts were centered on the person mean, and between-person parts on the grand mean (Curran & Bauer, 2011). We predicted the change goals (level 1 variable; different scores for each person and each character strength) by well-being, the between-parts of character strengths (level 2 variables; different scores for each person), and within-person parts of character strengths (level 1 variable; different scores for each person and each character strength), and their interaction with well-being while allowing for a random intercept for the participants and a random slope for the within-person part of character strengths (see Online Supplementary Equation 1 for model equation).

For examining research question 5 (examining what attributes of character strengths predict change goals), we also conducted multilevel models. We analyzed the attributes of character strengths that might contribute to change goals, taking into account their associations with morality (Sun & Goodwin, 2020; Thielmann & de Vries, 2021), their connection to well-being (Hudson & Fraley, 2016a), their normative levels, and the extent to which they are valued. These attributes were all on the level of character strengths (i.e., scores varying between strengths, but not between participants) and were considered in both studies. In Study 2, we additionally included participants’ ratings of the subjective contribution of each character strength to well-being (individual-level data, different scores for each participant). For this purpose, we again analyzed the data in the “long” format and predicted the change goals (level 1 variable; different scores for each person and each character strength) by each of the above-mentioned attributes of character strengths (level 2 variables; different scores for each character strength) separately while controlling for the individual character strengths trait levels and the individual well-being (level 2 variables; different scores for each person) in a multilevel model with random intercepts for both the participants and the type of character strengths (see Online Supplementary Equation 2 for model equation).

For all multilevel models, we report standardized regression weights (obtained by standardizing each variable before analysis; Lorah, 2018). For examining the explained variance in multilevel models, we report marginal (variance explained by fixed effects) and conditional *R*^2^ (variance explained by fixed and random effects; Nakagawa & Schielzeth, 2013).

For our analyses, we used R (Version 4.2.1; R Core Team, 2022) and the R-packages *dplyr* (Version 1.1.0; Wickham et al., 2023), *ggforestplot* (Version .1.0; Scheinin et al., 2023), *ggplot2* (Version 3.4.1; Wickham, 2016), *likert* (Version 1.3.5; Bryer & Speerschneider, 2016), *lme4* (Version 1.1.31; Bates et al., 2015), *lubridate* (Version 1.9.2; Grolemund & Wickham, 2011), *papaja* (Version .1.1; Aust & Barth, 2022), *parameters* (Version .20.2; Lüdecke et al., 2020), *psych* (Version 2.2.9; Revelle, 2022), *purrr* (Version 1.0.1; Wickham et al., 2023), *pwr* (Version 1.3.0; Champely et al., 2020), and *sjPlot* (Version 2.8.14; Lüdecke, 2021).

## Results

### Which character strengths do people want to change?

**Figure 1.**
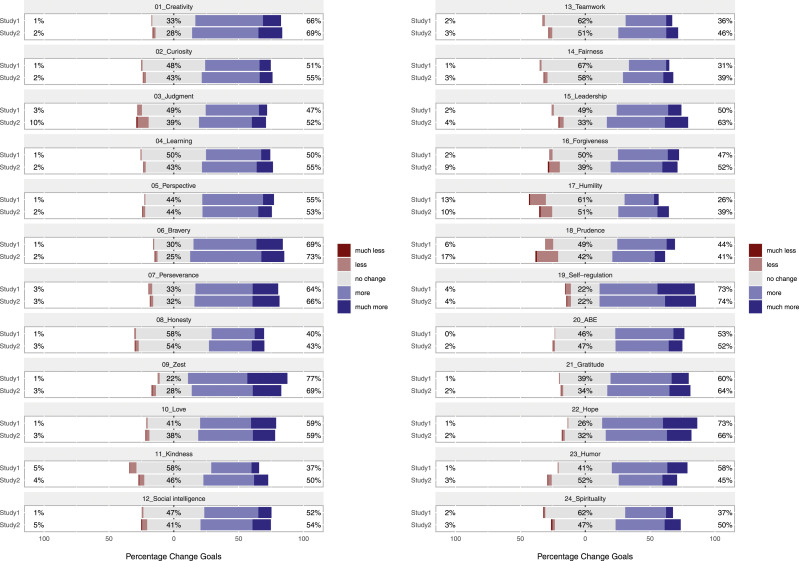
Frequency of change goals. *Note.* Learning = love of learning, ABE = appreciation of beauty and excellence. Percentages in the center (gray) area denote the proportion of participants who indicated “no change,” percentages on the left the proportion of participants who indicated “much less” or “less,” and percentages on the right those who indicated “more” or “much more.” Character strengths are sorted according to Peterson and Seligman (2004) by their assigned overarching virtue.

**Figure 2.**
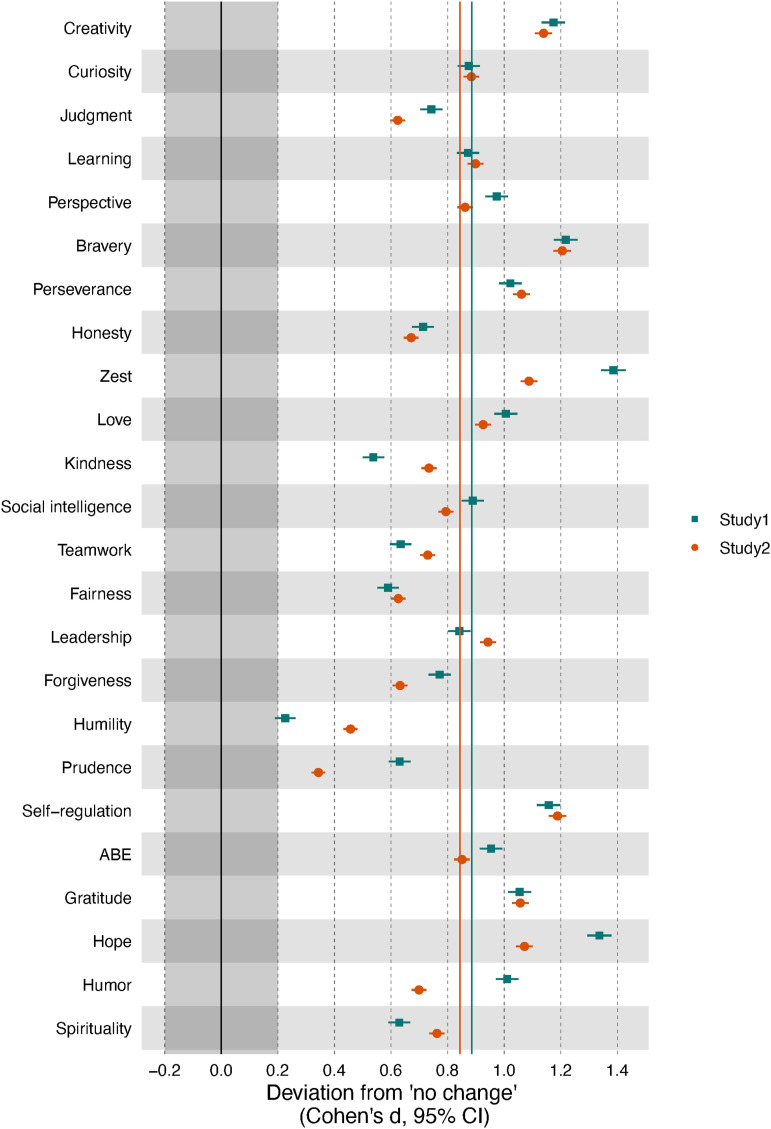
Comparison of average change goals with “no change.” *Note*. Learning = love of learning, ABE = appreciation of beauty and excellence. The x-axis denotes the difference from the mean of the respective change goal from zero (= no change desired) in Cohen’s d metric. Colored straight lines represent the average desire to change across all character strengths across each study. The grayed-out area indicates effects that were below the smallest effect size of interest.

Percentages of individuals who wanted lower levels, higher levels, or the same level of each character strength are given in Figure 1. The mean of the change goals and the comparison with the value 0 (= no change desired) are given in Figure 2 (see Online Supplementary Table S1A for means and standard deviations; https://osf.io/2xfyq/).

Figure 2 shows that, on average, people wanted to increase all character strengths in both studies: The difference of the change goal with “no desire to change” was at least of medium effect size, except for humility in both studies and prudence in Study 2 (small effect size). When comparing change goals among character strengths across both studies (see Table S1A), we found the desires to change creativity, bravery, perseverance, zest, love, self-regulation, gratitude, and hope to be stronger than on average across all character strengths (by at least a small effect). In contrast, the strengths of judgment, honesty, kindness, teamwork, fairness, forgiveness, humility, prudence, and spirituality showed comparatively smaller desires for change (differences of at least a small effect size). Overall, results from Study 1 and Study 2 converged well: Both the overall average desire to change (Study 1: Cohen’s *d* = .62; Study 2: Cohen’s *d* = .64) and the patterns of change goals (*r*_s_[23] = .86) were highly similar.

Further, a highly similar pattern emerged when considering the strengths selected for training in Study 1 (see Supplementary Figure S1). The character strengths of self-regulation (selected by 16% of participants), zest (14%), hope (12%), love (10%), perseverance (8%), humor (8%), and bravery (6%) were chosen more frequently for the training than what would be expected if all character strengths were selected equally (i.e., 100%/24 = 4.2%). Conversely, teamwork, fairness (both selected by <1%), love of learning, kindness, humility, prudence, appreciation of beauty and excellence, spirituality (all 1%), as well as curiosity, judgment, social intelligence, gratitude (all 2%), and honest (3%) were chosen less frequently.

### How do character strengths change goals relate to character strengths levels?

**Figure 3.**
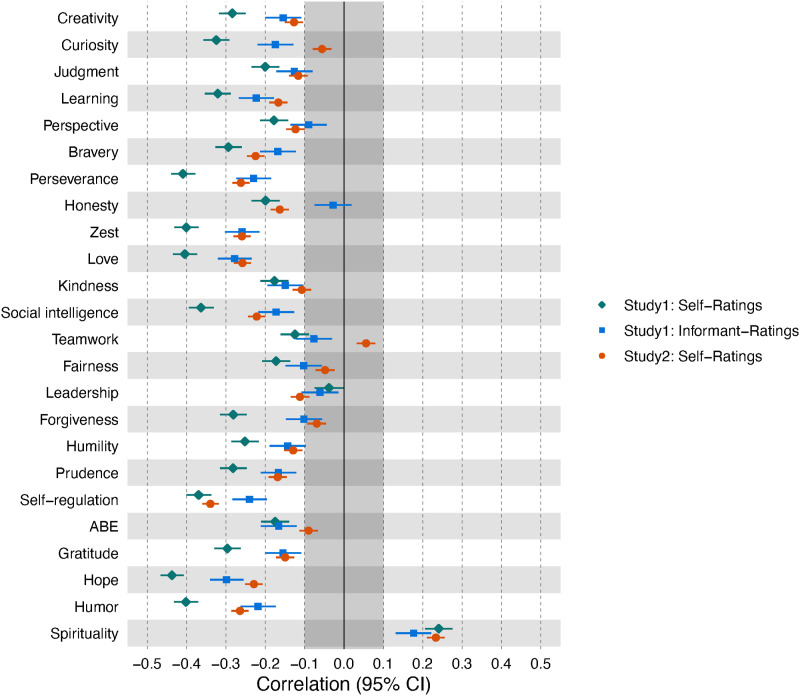
Correlations between character strengths levels and character strengths change goals in Studies 1 and 2. *Note*. Learning = love of learning, ABE = appreciation of beauty and excellence. Self-ratings = correlations between self-rated character strengths levels and self-rated change goals. Informant-ratings: correlations of informant-rated character strengths levels and self-rated change goals. The grayed-out area indicates effects that were below the smallest effect size of interest.

Correlations between character strength traits (self- and informant-ratings) and corresponding change goals (self-ratings) are given in Figure 3 (see Online Supplementary Figures S1A and S2 for off-diagonal correlations). Overall, for almost all character strengths, a lower trait level was related to a higher desire for change (i.e., a negative correlation between character strengths levels and the associated change goals) with at least a small effect size. The exceptions were spirituality (positive relationship in both studies) and several strengths that yielded no relationship in one of the studies (Study 1 self-ratings: leadership; Study 1 informant ratings: perspective, honesty, teamwork, and leadership; Study 2 self-ratings: curiosity, teamwork, fairness, forgiveness, and appreciation of beauty and excellence). Although the overall patterns of self-ratings in both studies and informant ratings were highly similar (self-ratings in Studies 1 and 2: *r*_s_[23] = .83, self-ratings and informant-ratings in Study 1: *r*_s_[23] = .91, self-ratings in Study 2 and informant-ratings in Study 1: *r*_s_[23] = .76), the relationships were generally smaller in Study 2 (mean *r* = −.14) and for informant ratings (mean *r* = −.15), than for self-ratings in Study 1 (mean *r* = −.26).

Additional preregistered analyses in Study 1 are reported in Online Supplementary Tables S2 (linear and quadratic relationships between change goals and character strengths) and S3 (relationships of character strengths traits with the desire to change across all character strengths, regardless of the direction).

### How do character strengths change goals relate to age?

**Figure 4.**
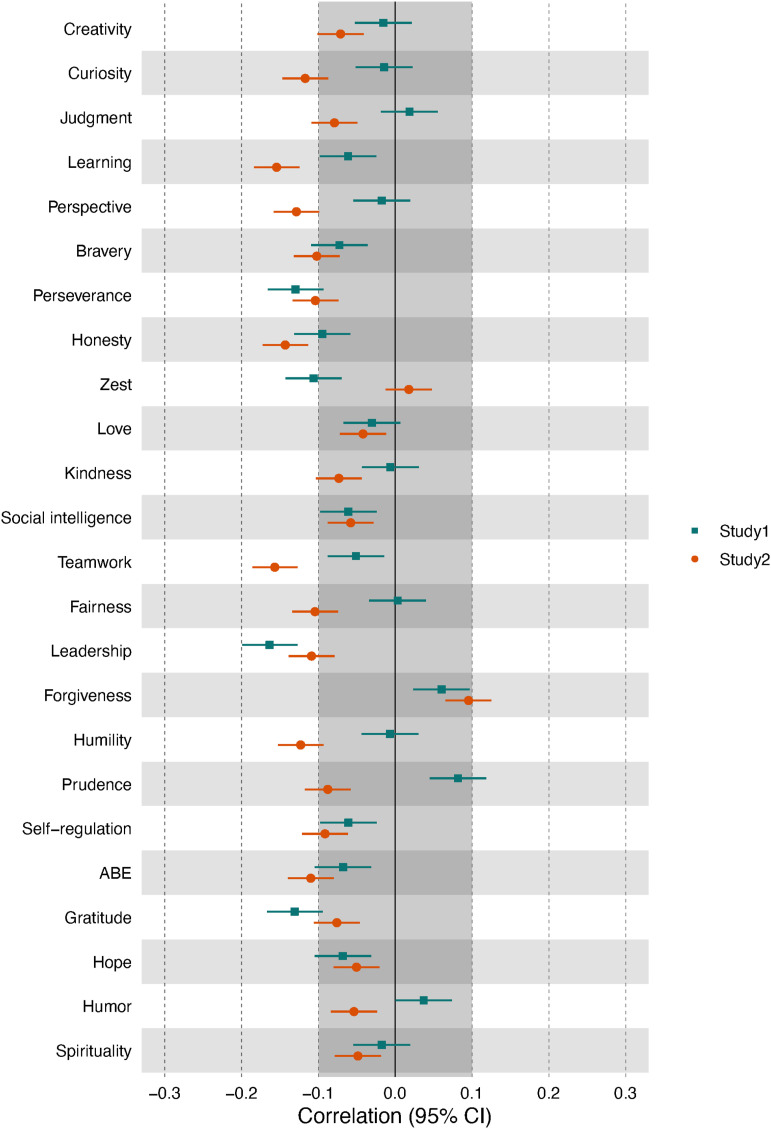
Correlations of change goals with age, adjusted for trait levels. *Note*. Learning = love of learning, ABE = appreciation of beauty and excellence. The grayed-out area indicates effects that were below the smallest effect size of interest.

To examine character strengths’ associations with age, we analyzed the partial correlations of character strengths change goals with age, corrected for character strengths traits^3^ (Figure 4; see Online Supplementary Figure S4 for zero-order correlations).

While for most character strengths change goals, younger participated reported a stronger desired for change than older participants, most of the relationships with age were of negligible size. Only perseverance and leadership yielded effects of at least small size (i.e., |*r*| ≥ .10) in both studies. Thus, younger participants reported a stronger desire to increase perseverance and leadership than older participants. Further, two additional strengths yielded negative relationships of small effect size in Study 1 and nine additional strengths in Study 2. Both studies converged on a near-zero average relationship with age (Study 1: mean *r* = −.04, Study 2: mean *r* = −.08), and the pattern of relationships showed some similarity across the studies, *r*_s_[23] = .19.

### How do character strengths change goals relate to well-being?

**Figure 5.**
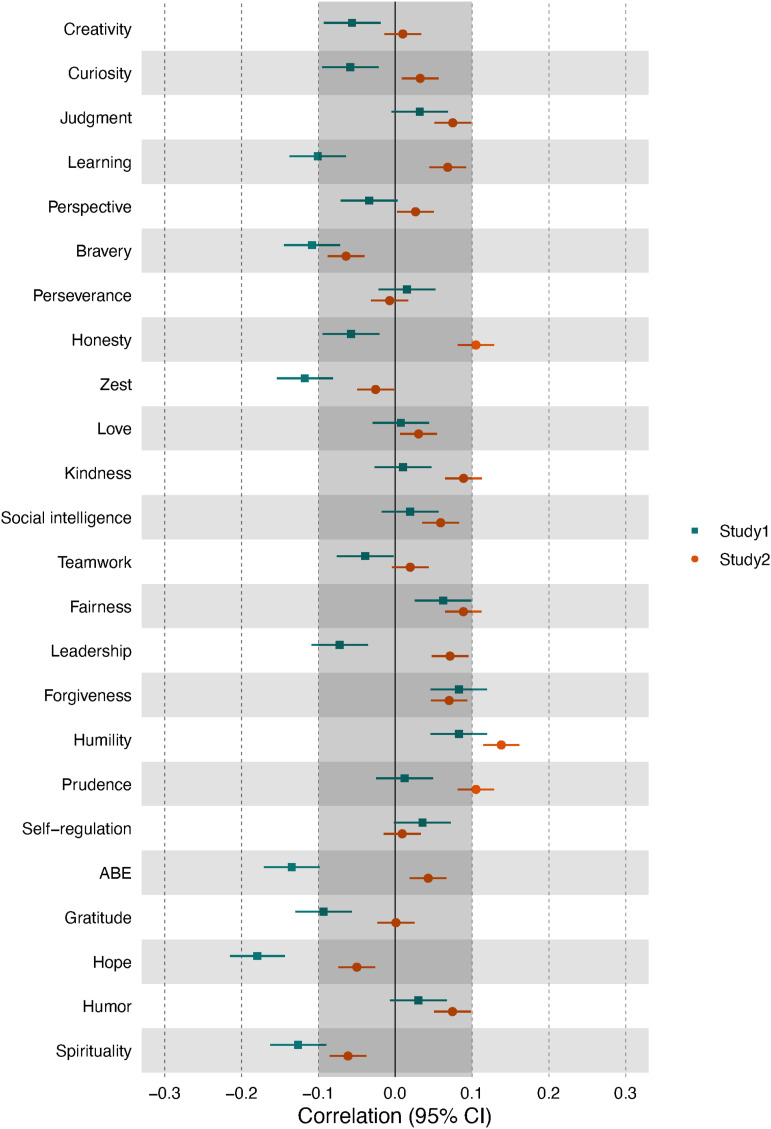
Correlations of change goals with well-being, adjusted for trait levels. *Note*. Learning = love of learning, ABE = appreciation of beauty and excellence. The grayed-out area indicates effects that were below the smallest effect size of interest.

For studying the relationships of character strengths with well-being, we again computed partial correlations adjusted for the trait levels (Figure 5; see Online Supplementary Table S4A for zero-order correlations).

Again, the findings from both studies showed some similarities, although different strengths surpassed our pre-defined threshold of a small effect. In Study 1, six strengths yielded a negative relationship: A stronger desire to increase hope, spirituality, appreciation of beauty and excellence, zest, bravery, and love of learning was related to lower well-being after adjusting for trait levels, while there were no positive relationships. In Study 2, those with higher levels of well-being reported a stronger desire to change honesty, humility, and prudence, while there were no negative relationships. No association exceeded the pre-defined effect size threshold across both studies. Average relationships with well-being were close to zero (Study 1: mean *r* = −.03, Study 2: mean *r* = .04), and the pattern of relationships was similar across the studies, *r*_s_[23] = .56.

When examining the correlations of change goals with different domains of well-being in Study 1, the overall pattern of associations was highly similar to the relationships reported above for the well-being mean score in Study 1 (see Online Supplementary Table S4B). Nonetheless, depending on the specific domain of well-being, different character strengths yielded relationships with well-being of at least a small effect size. The strongest specific associations were found for the desire to increase hope with optimism (*r* = −.30), positive affect (*r* = −.30), negative affect (*r* = −.29), and life satisfaction (*r* = −.22), and for the desire to increase zest with engagement (*r* = −.24).

### Do relationships of levels of character strengths and character strengths change goals depend on well-being levels?

**Figure 6.**
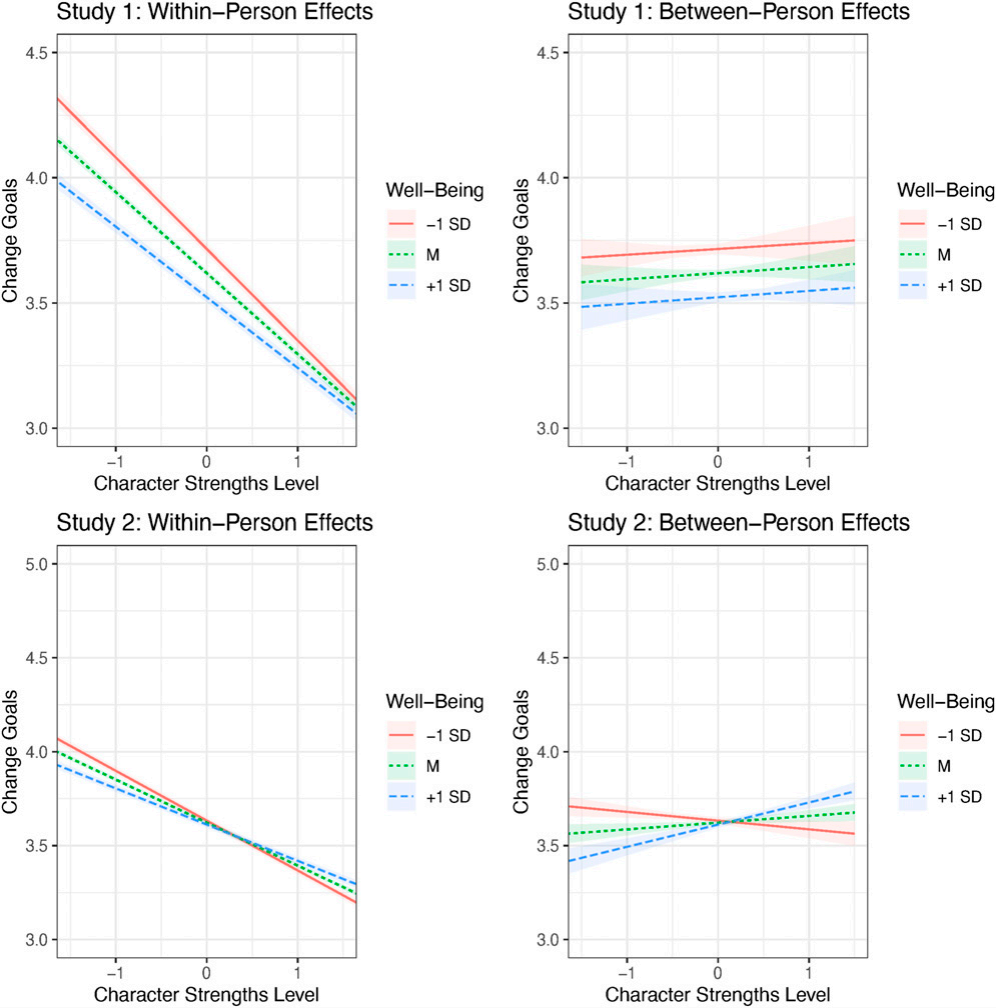
Interaction between character strengths traits and well-being in the prediction of change scores (standardized fixed effects). *Note.* The character strength levels are presented as z-scores, and the shaded areas represent the corresponding 95% confidence intervals. The interaction of well-being with between-person effects in Study 1 was not significant, while in Study 2, the between-person effects were significant for well-being values smaller than −.85 and larger than −.08. The within-person effects were significant for all observed values of well-being.

For a more thorough analysis of the interplay of character strengths traits, change goals, and well-being, we examined the interaction between trait levels of character strengths and well-being in the prediction of change goals.^4^ We predicted the change goals simultaneously by well-being, the between- and within-person parts of character strengths levels, and the interaction between well-being and the between- and within-person parts of character strengths in a multilevel model with random intercepts for the participants (see Table 1 and Figure 6).

**Table 1.** Prediction of Change Goals by Individual Trait and Well-Being Levels, and Their Interaction (Predictors Entered Simultaneously, Standardized Fixed Effects With 95% Confidence Intervals).

	Study 1		Study 2	
Predictors	Estimate	*df*	Estimate	*df*
Fixed effects				
Intercept	−.00 [−.02, .02]	2783.01	−.02 [−.04, −.01]	6641.87
Well-being	**−.13 [**−**.15;** −**.11]**	2783.02	−.01 [−.03; .00]	6659.67
CS levels (between-person part)	.01 [−.01; .03]	2782.97	.02 [.00; .03]	6612.83
CS levels (within-person part)	**−.24 [**−**.24;** −**.23]**	2549.27	**−.19 [**−**.20;** −**.18]**	6113.07
Well-being × CS levels (between-person part)	.00 [−.01; .01]	2782.97	**.04 [.03; .05]**	6610.92
Well-being × CS levels (within-person part)	**.03 [.02; .04]**	2253.79	**.04 [.03; .04]**	5657.38
Random effects				
σ_*Intercept*_^2^	.07		.15	
σ_*Slope*_^2^	.05		.09	
σ_ε_^2^	.41		.40	
cor_intercept-slope_	−.01		.17	
*N*_Persons_/*N*_Observations_	2,787/66,888		6,615/158,113	
Marginal R^2^/conditional R^2^	.08/.24		.04/.36	

*Note*. CS = character strengths. σ_*Intercept*_^2^ = variance of random intercept for individuals, σ_*Slope*_^2^ = variance of random slope for within-person part of character strengths, σ_ε_^2^ = residual error variance. Confidence intervals not including zero are highlighted in bold.

Table 1 shows that across both studies, there was an interaction between the within-person parts of character strengths and well-being (Figure 6, left side): The association between character strengths levels and change goals decreased with well-being, suggesting that those with lower well-being are more committed to changing the strengths they possess to a lesser degree (relatively to their personal strength profile) than those with higher well-being. Study 2 additionally revealed an interaction between the between-person aspects of character strengths and well-being (Figure 6, right side). This suggests that individuals with higher well-being tend to report more pronounced change goals when they possess higher overall levels of character strengths. Overall, the two studies converged well in suggesting that the strongest effect was obtained for the within-person parts of character strengths levels, although some minor differences were observed (based on a comparison of confidence intervals). Effects of well-being levels were only observed in Study 1, effects of within-person parts of character strengths levels were slightly higher in Study 1, and the interaction of between-person parts with well-being was only observed in Study 2.

### Do the attributes of a trait predict change goals?

We analyzed the attributes of character strengths that might contribute to change goals, taking into account their associations with morality (Sun & Goodwin, 2020; Thielmann & de Vries, 2021), their connection to well-being (Hudson & Fraley, 2016a), their normative levels, and the extent to which they are valued, while controlling for individual character strengths trait levels, and individual well-being levels. When analyzing each attribute separately, results confirmed earlier findings by Sun and Goodwin (2020) that the desire to change a trait declined with the traits’ associations with morality: β = −.14 [−.27; −.02]/ −.09 [−.16; −.02] (Study 1/Study 2). At the same time, the higher a trait’s association with well-being, the stronger the desire to change this trait: β = .23 [.13; .32]/.11 [.05; .18]. We found no effects for normative trait levels .10 [−.03; .23]/ −.04 [−.12; .04], while the valuing of a trait in the society only had an effect in Study 2 (Study 1: β = .04 [−.10; .18]/Study 2: .09 [.02; .16]). The best predictor—besides trait levels in the respective strength—was a strength’s subjective contribution to well-being .18 [.17; .18] (only assessed in Study 2). Full results of separate regressions are given in Online Supplementary Table 5. Overall, the two studies agreed in terms of the direction and size of the effects, and no differences between the coefficients of the attributes (based on a comparison of confidence intervals) were observed. However, the negative associations of change goals with trait levels were stronger in Study 1, while individual well-being was positively related to change goals only in Study 2.

When regressing all attributes of character strengths simultaneously on change goals (Table 2), only the associations with well-being value remained a significant predictor (above the individual character strengths traits scores and well-being scores) across both studies. In Study 2, change goals were predicted best by the subjective contribution of a character strength to well-being.

**Table 2.** Prediction of Change Goals by Individual Trait and Well-Being Levels, and Normativity, Desirability, and Associations With Morality and Well-Being (Predictors Entered Simultaneously, Standardized Fixed Effects With 95% Confidence Intervals).

	Study 1		Study 2	
	Estimate	*df*	Estimate	*df*
Intercept	.00 [−.09, .09]	20.29	.00 [−.05, .05]	21.35
Individual character strengths traits	**−.31 [**−**.32,** −**.30]**	66,878.52	**−.24 [**−**.25,** −**.24]**	154,630.79
Individual well-being (mean score)	−.02 [−.04, .00]	2,927.89	**.03 [.02, .05]**	6,749.80
Association with morality	−.06 [−.18, .05]	19.00	−.04 [−.10, .02]	19.00
Association with well-being	**.20 [.09, .31]**	19.00	**.08 [.02, .14]**	19.00
Character strengths valuing	.08 [−.02, .18]	19.00	.06 [.00, .12]	19.00
Character strengths normative level	.08 [−.02, .17]	19.05	.02 [−.04, .08]	19.01
Subjective contribution to well-being	–	–	**.18 [.17, .18]**	153,876.20
Random effects				
σ_*Person*_^2^	.16		.24	
σ_*Strength*_^2^	.04		.01	
σ_ε_^2^	.69		.66	
*N*_Persons_/*N*_Strengths_/*N*_Observations_	2,787/24/66,888		6,558/24/155,492	
Marginal R^2^/conditional R^2^	.15/.34		.09/.34	

*Note*. Association with morality = correlations of character strengths traits with self-ratings of moral character (data from Wagner et al., 2021); association with well-being = correlations with well-being mean score, derived from the present study, character strengths valuing = valuing ratings of character strengths in Western Europe (Study 1) and North America (Study 2; reported in Pievsky & McGrath, 2022), character strengths normative levels = mean levels of character strengths reported in Ruch et al., (2010; Study 1) and McGrath and Wallace (2021; Study 2). Subjective contribution to well-being = participant’s rating of how strong each strength contributes to their well-being. σ_*Person*_^2^ = variance of random intercept for individuals, σ_*Strength*_^2^ = variance of random intercept for type of character strength, σ_ε_^2^ = residual error variance. Confidence intervals not including zero are highlighted in bold.

## Discussion

The present set of two studies examined character strengths change goals and their relationships to individual differences such as age, well-being, and self- and informant-rated character strengths levels. Results suggested that participants expressed a desire to increase all 24 character strengths, especially those strengths they (and others) perceive to be lacking. Although older people tended to report lower intentions to change most of their character strengths, the associations were mostly negligible in size. While well-being only showed marginal effects on change goals, an individual’s well-being level moderated the relationships between character strengths traits and change goals, with stronger associations between character strengths traits and change goals for those with lower well-being. Further, when studying additional predictors of change goals, the results suggested that a strength’s (subjective) contribution to well-being is of particular relevance.

### Who wants to change which traits?

Overall, we found that participants wanted to increase all 24 character strengths on average, and only a few people desired to decrease these traits. At the same time, both the proportion of people desiring positive change and the average extent of desired change varied considerably across the character strengths. The character strengths of creativity, bravery, perseverance, zest, love, self-regulation, gratitude, and hope yielded the greatest desire for positive change, whereas the desire to increase judgment, honesty, kindness, teamwork, fairness, forgiveness, humility, prudence, and spirituality was markedly lower but still positive. In concert, these findings corroborate earlier notions that most people want to change some aspects of their personality (e.g., Miller et al., 2019) and support Peterson and Seligman’s (2004) idea that all 24 character strengths represent positively valued, desirable traits.

### Change goals and trait levels

Most character strengths change goals were negatively related to trait levels of the same strengths, with the exception of spirituality, which yielded a positive relationship. Effect sizes were, on average, of medium size but varied among strengths and between studies. This is in line with earlier findings reporting a generally negative relationship between the level of a trait and the desire to change it (e.g., Hudson & Fraley, 2016a; Hudson & Roberts, 2014; Miller et al., 2019; Robinson et al., 2015; Thielmann & de Vries, 2021). The results also suggest that the character strength of spirituality is a special case that contradicts the general rule: The more spiritual a person, the more they want to further increase their spirituality, or, said differently, people with low levels of spirituality rarely want to become more spiritual.

The character strength of spirituality overlaps with some facets of the five-factor model dimension of openness, specifically with openness to values (McGrath et al., 2020). For openness, the relationships between the trait level and change goal were inconsistent across studies (Thielmann & de Vries, 2021). Our findings indicate that while other character strengths that share similarities with openness facets, such as creativity and appreciation of beauty and excellence, exhibit the typical negative association between trait level and desire to change, spirituality shows a positive association. This suggests the possibility that facets of openness may differ in their trait level-change goals, potentially explaining the overall inconsistent or zero relationship.

### Change goals and well-being

No character strength change goals showed consistent relationships with well-being of at least a small size across both studies. Across both studies and all character strengths, there was no relationship between change goals and well-being after accounting for character strengths trait levels. Thus, our findings are in line with earlier findings, which did not find relationships between change goals and well-being after controlling for trait levels, except for conscientiousness (Hudson & Roberts, 2014; Quintus et al., 2017). The present study’s findings corroborate the notion that low well-being does not generally result in a higher motivation for change.

For the relationships with different facets of well-being, the overall pattern was highly similar to the results for global well-being. At the same time, some specific, meaningful relationships emerged: For example, people with low levels in the well-being dimensions of engagement especially desired to increase zest, those with low levels in mastery desired to increase love of learning, and those with low levels in meaning aimed at fostering hope and spirituality, and those with low levels in optimism aimed at fostering the character strength of hope. Thus, several of those specific relationships between strength change goals and well-being dimensions converge with findings on the relationships between character strengths and the respective well-being dimensions (Hausler et al., 2017b; Wagner et al., 2020). Building on these results, one could conclude that people are also guided by the desired outcome when deciding which characteristics to change. Thus, dissatisfaction with specific aspects of well-being might be considered a motivator for change in specific directions, which is in line with previous findings on dissatisfaction with life domains (Hudson & Roberts, 2014).

### Relationships between change goals and trait levels moderated by well-being

In line with previous research (e.g., Costantini et al., 2020; Hudson & Fraley, 2016a; Hudson & Roberts, 2014; Miller et al., 2019; Quintus et al., 2017; Robinson et al., 2015; Sun & Goodwin, 2020), we found that people were generally most interested in increasing the character strengths they perceive as lacking. We also found that, when controlling for trait levels, the relationships between change goals and well-being were close to zero. Even though well-being did not seem to drive change goals generally, we found that its interaction with trait levels was relevant for the desire to change: Consistently across both studies, we found that those with lower well-being are more interested in changing the strengths they relatively lack compared to those with higher well-being. That is, unhappy individuals tend to endorse compensatory goals (Reisz et al., 2013) more strongly; they seek to improve upon what they may perceive as weaknesses. Conversely, happy individuals show this tendency less strongly.

In Study 2, we additionally found that happier people reported higher change goals overall when they possessed higher trait levels of character strengths. That is, they tended to aspire to complementary goals (Reisz et al., 2013). In line with Kaiser et al. (2020), we suggest that high well-being does not necessarily result in a decreased desire for change in general but can also be seen as indicative of resources that enable individuals to aspire to goals and direct the desired change toward areas that are already well-developed.

### Change goals and age

Overall, older people tended to have less pronounced change goals than younger people, but most relationships were negligible in size. The most consistent negative relationships were found for leadership and perseverance. This is in line with earlier research on big five-related change goals, which also reported a stronger desire for change in younger samples (Hudson & Fraley, 2016b; Quintus et al., 2017). Given that older people also tend to report higher trait levels of most character strengths (Heintz & Ruch, 2022), one possible explanation is that over the life course, people are somewhat successful in changing their character in the desired direction, and therefore, trait levels increase, and the motivation for further change decreases. Cornwell and colleagues (2022) also reported a negative relationship between age and growth motives but also showed that those people who did not follow this trend (i.e., those who maintained their growth motives) reported *lower* levels of well-being. The authors conclude that a weaker desire for change can also be adaptive, indicating that the intended goals have been achieved. However, another possibility would be that as people age, they become more accepting of their personalities and, therefore, do not strive for further improvement.

While not exceeding small effect sizes, there were some noteworthy differences among character strengths, and for some strengths, namely, prudence and forgiveness, the desire to change these strengths *increased* with age. This is in line with earlier ideas that with increasing age, different developmental tasks become more relevant, which might require different traits and thus drive personality development (Hutteman et al., 2014). For example, Baumann et al. (2020) reported that for some strengths, such as prudence, the relationship to well-being increases with age and with age-specific developmental tasks. Among the retired, prudence (and other strengths) played a more important role than among the employed. This line of thought would also explain why career-related strengths such as leadership and perseverance yielded the strongest negative relationships with age; however, it fails to account for the negative relationship with gratitude or zest.

### Which attributes of traits predict change goals?

When examining which attributes of character strengths contribute to change goals above individual trait levels and well-being levels, our results support previously raised notions (e.g., Sun & Goodwin, 2020) that people have lower desires to change moral traits compared to less moral traits. However, even stronger relationships were found between change goals and a trait’s relationship with well-being. In contrast, normative levels of traits and the national valuing of traits were unrelated to change goals. At least for character strengths, the more moral traits also tended to show smaller relationships with well-being (*r*_s_[23] = −.32/−.31 in Studies 1 and 2). Subsequently, a trait’s relationship to well-being remained the sole significant predictor of change goals when considering all mentioned attributes of traits simultaneously. This finding was also apparent for the relationships of change goals with well-being: The pattern of relationships bears a considerable resemblance with the pattern of associations between character strengths traits and well-being (*r*_s_[23] = −.50/−.73 in Studies 1 and 2). Thus, overall, the trait’s relationship with well-being seemed to be the most important driving force for the desire to change it.

In sum, our results provide some support for Sun and Goodwin’s (2020) conclusion that “moral considerations take a back seat when it comes to self-improvement” (p. 243). However, they also offer evidence for one of the explanations they provided for their findings: When thinking about which traits they want to change, people might be primarily driven by the expected individual outcomes. Thus, people (and especially unhappy people) might especially strive toward fostering those traits that promise the strongest gains in well-being. In an extension of Luhmann and Hennecke’s (2017) proposition, this would suggest that unhappiness is a motivator of *directed* change toward more well-being. Of course, this idea entails that people have a rather accurate idea of which traits could contribute to well-being. Findings from Study 2 suggest that people’s notions about the contributions of the trait to well-being are indeed important drivers of their goals for volitional personality change.

This conclusion aligns well with recent findings on the question of why people want to become more moral. Sun et al. (2023) found that the extent to which people believe that changes would benefit their well-being was the best predictor of their motivation to increase moral traits. Our findings support their interpretation that the belief that one will become happier as a result of changing one’s personality traits is an important mechanism to be considered in studying volitional personality change.

### Theoretical and practical implications

From a theoretical standpoint, the present findings suggest that when studying change goals, it is important to consider not only the differences between individuals but also the differences between traits. Previous studies identified a trait’s contribution to morality (Sun & Goodwin, 2020) or its social desirability (Thielmann & de Vries, 2021) as relevant predictors of change goals. The present study corroborated this finding but also suggests that the previously reported negative association of change goals with a trait’s morality (Sun & Goodwin, 2020) could also be, at least partially, explained by a trait’s association with well-being. Additional attributes could be of relevance for the study of change goals, including the perceived normativity or valuing of traits (e.g., in one’s culture, community, or peer group). While the present study only used population-average estimations of these attributes, subjectively perceived levels would presumably be more relevant (as found for population-level vs. subjective contributions to well-being in Study 2).

From an applied standpoint, the findings of the present manuscript could have practical implications for the design of interventions. One commonly used individualized approach in strengths-based interventions is to encourage individuals to focus on their highest strengths (Ghielen et al., 2018). However, previous research has shown that outcomes such as increased happiness, decreased depression, and enjoyment of the intervention are not affected by whether individuals work on their strengths or weaknesses (Proyer et al., 2015). The present study suggests that people report a stronger desire to change the character strengths they perceive as lacking, which may indicate that it is more effective to focus on developing one’s “weaknesses” or lower strengths to increase motivation without diminishing other positive outcomes. Another commonly used approach is to provide individuals with a pre-defined set of character strengths to work on. Previous research by Proyer et al. (2013) and Ruch et al. (2020) has shown that training strengths that are strongly associated with well-being lead to greater increases in well-being than training strengths with weaker associations. The present study suggests that individuals may also be more motivated to work on character strengths that have a stronger relationship with well-being. In addition, when designing interventions, it may be worthwhile to consider the specific attributes of the traits studied in this research. For instance, in psychoeducational components of interventions, it could be beneficial to highlight a strength’s association with well-being (or morality or social desirability) in order to increase participants’ engagement in the intervention.

### Limitations and directions for future research

Several additional limitations of the present study must be mentioned. First, given the obvious cultural variations in the desire to change personality traits (e.g., Baranski et al., 2021) and in the valuing of character strengths (Pievsky & McGrath, 2022), our results cannot readily be generalized beyond German-speaking and English-speaking populations. Second, our measures of character strengths change goals were developed ad-hoc, though they were closely based on both existing measures of perceived changes in character strengths and existing measures of personality change goals, such as a 1-item measure already used by Thielmann and de Vries (2021). However, different methods to assess personality change goals have been found to converge only moderately (Miller, 2022). It would, therefore, be necessary to further validate this measure alongside alternative measures of character strengths change goals.

Third, the present samples differed from a general population sample in several main aspects. Most importantly, participants in Study 1 self-selected into an online positive psychology intervention study aimed at fostering well-being, indicating a high pre-existing motivation for change. This self-selection may have influenced the results, as individuals who are more motivated to change may have more pronounced change goals. Participants in Study 2 were recruited from a website where they completed a character strengths measure to obtain individual feedback. Consequently, these people might be more motivated to reflect on their personality and potential desired changes than the general population, representing a potential source of bias. When comparing the results obtained in the two samples, we observed no differences in overall levels of change goals, even though one could assume that people signing up for an online intervention would be more inclined to change. We did, however, observe some differences that might be interpreted as participants in Sample 1 being more self-reflective; namely, participants in Sample 1 showed a stronger negative relationship between character strengths traits and change goals than participants in Sample 2. In addition, both samples were also somewhat imbalanced in gender distribution (79% and 67% women), which represents a potential constraint on the generality of the results. However, we did not observe any noteworthy gender differences in our analyses (see Online Supplementary Tables S6–S10).

Fourth, we used measures of different lengths to assess character strengths (Study 1: 240 items for self-report, 120 items for informant report; Study 2: 96 items). This difference is inarguably one of the reasons we found the correlations between character strengths traits and change goals to be lower for the informant-reported character strengths in Study 1 and self-reported character strengths in Study 2 than for the self-reported character strengths in Study 1. Fifth, we used global estimates of the strengths’ association with morality as well as global ratings of their normativity and national valuing. While we think that these population-level estimates can serve as rough indicators for an initial test of these ideas, future studies might directly ask participants about a trait’s subjectively perceived moral value. Using person-level indicators could offer a more nuanced study of the contribution of trait attributes to change goals. Finally, all our findings were based on cross-sectional data, and all discussions of directions of effects (e.g., whether well-being drives the desire to change or vice versa) or effects related to aging remain speculative because our study design does not allow disentangling developmental and cohort effects.

In the present study, we did not assess goals to change the five-factor dimensions of personality, so we cannot study the relationships between goals to change personality (as defined by the five-factor or HEXACO model) and character strengths. Thus, the incremental validity of goals to change character strengths beyond goals to change personality traits of the five-factor model is yet to be established. Future research should directly contrast goals to change five-factor model dimensions and character strengths to be able to inform this question, which the present study cannot answer.

### Conclusions

We conclude that (I) people generally want to increase all 24 character strengths but that these desires are especially pronounced for strengths such as zest, hope, self-regulation, bravery, creativity, perseverance, love, gratitude, and hope. As has been reported for non-valued personality traits, (II) people especially want to increase those character strengths that they relatively lack, both in their self-perception and perceptions by close others. One exception was spirituality, where individuals with higher trait expressions indicated a higher intention to change. Further, (III) most associations of change goals with age and well-being were negligible in size after controlling for character strengths trait levels. Thus, people with lower well-being may not generally want to change more—but when they do want to change, they tend to do so differently than people with high well-being: Less happy individuals focus on developing new character strengths (i.e., the ones they have lower levels of) more strongly than happier individuals. Thus, well-being does not necessarily drive the desire to change but rather guides the direction of an existing desire to change. Finally, (IV) whether someone wants to change a trait not only depends on one’s standing on this trait but is also related to trait-specific attributes, especially a trait’s relationship to well-being. This finding suggests that people strive to promote the traits that benefit well-being most.

## Data Availability

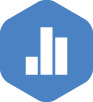
 
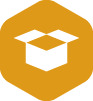
 
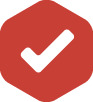
 The raw data of Study 1 and the code underlying all analyses, supplementary Tables and Figures, and a document detailing the deviations from the preregistrations of Studies 1 and 2 are available from the project’s OSF page (https://osf.io/2xfyq). All study materials of Study 1 are included in the code book. Due to the data privacy policy of the institute that collected the data for Study 2, the data, unfortunately, cannot be shared.

## Notes

1. We deviated from the preregistration in not calculating all further analyses with both ratings of change goals and the character strengths selected for training, since the measurement of the latter was directly dependent on the former (see the Instruments section).

2. This was first suggested by a reviewer and became even more justified given the large sample size in Study 2.

3. We deviated from the preregistered procedure and focused mainly on partial correlations (instead of zero-order correlations) because of the strong relationships of change goals with traits. We were interested in whether potential age-related differences are not simply due to age-related differences in trait levels which are small yet present according to a meta-analysis (Heintz & Ruch, 2022). In addition, controlling for trait levels is consistent with previous research on the topic (e.g., Sun & Goodwin, 2020) and makes our findings more comparable to previous work. At the same time, adjusting for the trait levels only had a negligible effect on the size of the relationships.

4. While we originally planned to conduct response surface analyses to examine this research question, we decided to prefer this method of analyzing the data in line with a suggestion by a reviewer.

## References

[bibr1-08902070231211957] Anglim J. Horwood S. Smillie L. D. Marrero R. J. Wood J. K. (2020). Predicting psychological and subjective well-being from personality: A meta-analysis. Psychological Bulletin, 146(4), 279–323. 10.1037/bul0000226 31944795

[bibr2-08902070231211957] Aust F. Barth M. (2022). papaja: Prepare reproducible APA journal articles with R Markdown [Manual]. https://github.com/crsh/papaja

[bibr3-08902070231211957] Baranski E. Gardiner G. Lee D. Funder D. C. (2021). Who in the world is trying to change their personality traits? Volitional personality change among college students in six continents. Journal of Personality and Social Psychology, 121(5), 1140. 10.1037/pspp0000389 34881959

[bibr4-08902070231211957] Bates D. Mächler M. Bolker B. Walker S. (2015). Fitting linear mixed-effects models using lme4. Journal of Statistical Software, 67(1), 1–48. 10.18637/jss.v067.i01

[bibr5-08902070231211957] Baumann D. Ruch W. Margelisch K. Gander F. Wagner L. (2020). Character strengths and life satisfaction in later life: An analysis of different living conditions. Applied Research in Quality of Life, 15(2), 329–347. 10.1007/s11482-018-9689-x

[bibr6-08902070231211957] Bleidorn W. Schwaba T. Zheng A. Hopwood C. J. Sosa S. S. Roberts B. W. Briley D. A. (2022). Personality stability and change: A meta-analysis of longitudinal studies. Psychological Bulletin. 148(7–8), 588–619. 10.1037/bul0000365 35834197

[bibr7-08902070231211957] Bruna M. O. Brabete A. C. Izquierdo J. M. A. (2019). Reliability generalization as a seal of quality of substantive meta-analyses: The case of the VIA Inventory of Strengths (VIA-IS) and their relationships to life satisfaction. Psychological Reports, 122(3), 1167–1188. 10.1177/0033294118779198 29848214

[bibr8-08902070231211957] Bryer J. Speerschneider K. (2016). likert: Analysis and visualization likert items [Manual]. https://CRAN.R-project.org/package=likert

[bibr9-08902070231211957] Champely S. Ekstrom C. Dalgaard P. Gill J. Weibelzahl S. Anandkumar A. Ford C. Volcic R. Rosario H. D. (2020). pwr: Basic Functions for power analysis (1.3-0). https://CRAN.R-project.org/package=pwr

[bibr10-08902070231211957] Cornwell J. F. M. Nakkawita E. Franks B. Higgins E. T. (2022). Motivation and well-being across the lifespan: A cross-sectional examination. The Journal of Positive Psychology. 18(5), 688–694. 10.1080/17439760.2022.2093787

[bibr11-08902070231211957] Costantini G. Saraulli D. Perugini M. (2020). Uncovering the motivational core of traits: The case of conscientiousness. European Journal of Personality, 34(6), 1073–1094. 10.1002/per.2237

[bibr12-08902070231211957] Curran P. J. Bauer D. J. (2011). The disaggregation of within-person and between-person effects in longitudinal models of change. Annual Review of Psychology, 62(1), 583–619. 10.1146/annurev.psych.093008.100356 PMC3059070 19575624

[bibr13-08902070231211957] Gander F. Hofmann J. Proyer R. T. Ruch W. (2020). Character strengths – stability, change, and relationships with well-being changes. Applied Research in Quality of Life, 15(2), 349–367. 10.1007/s11482-018-9690-4 PMC7250648 32457813

[bibr14-08902070231211957] Gander F. Wagner L. (2022). Character growth following collective life events: A study on perceived and measured changes in character strengths during the first wave of the COVID-19 pandemic. European Journal of Personality, 36(4), 466–482. 10.1177/08902070211040975 PMC13029417 41909813

[bibr15-08902070231211957] Gander F. Wagner L. Amann L. Ruch W. (2022). What are character strengths good for? A daily diary study on character strengths enactment. The Journal of Positive Psychology, 17(5), 718–728. 10.1080/17439760.2021.1926532

[bibr16-08902070231211957] Ghielen S. T. S. Woerkom M. van Meyers M. C. (2018). Promoting positive outcomes through strengths interventions: A literature review. The Journal of Positive Psychology, 13(6), 573–585. 10.1080/17439760.2017.1365164

[bibr17-08902070231211957] Gignac G. E. Szodorai E. T. (2016). Effect size guidelines for individual differences researchers. Personality and Individual Differences, 102, 74–78. 10.1016/j.paid.2016.06.069

[bibr18-08902070231211957] Grolemund G. Wickham H. (2011). Dates and times made easy with lubridate. Journal of Statistical Software, 40(3), 1–25. https://www.jstatsoft.org/v40/i03/

[bibr19-08902070231211957] Hausler M. Huber A. Strecker C. Brenner M. Höge T. Höfer S. (2017a). Validierung eines Fragebogens zur umfassenden Operationalisierung von Wohlbefinden [Validation of a holistic measure for the construct of well-being]. Diagnostica, 63(3), 219–228. 10.1026/0012-1924/a000174

[bibr20-08902070231211957] Hausler M. Strecker C. Huber A. Brenner M. Höge T. Höfer S. (2017b). Distinguishing relational aspects of character strengths with subjective and psychological well-being. Frontiers in Psychology, 8, 1159. 10.3389/fpsyg.2017.01159 28744245 PMC5504157

[bibr21-08902070231211957] Heintz S. Ruch W. (2022). Cross-sectional age differences in 24 character strengths: Five meta-analyses from early adolescence to late adulthood. The Journal of Positive Psychology, 17(3), 356–374. 10.1080/17439760.2021.1871938

[bibr22-08902070231211957] Höfer S. Hausler M. Huber A. Strecker C. Renn D. Höge T. (2020). Psychometric characteristics of the German values in action inventory of strengths 120-item short form. Applied Research in Quality of Life, 15(2), 597–611. 10.1007/s11482-018-9696-y 32457816 PMC7250639

[bibr23-08902070231211957] Hudson N. W. (2022). Lighten the darkness: Personality interventions targeting agreeableness also reduce participants’ levels of the dark triad. Journal of Personality, 91(4), 901–916. 10.1111/jopy.12714 35285028

[bibr24-08902070231211957] Hudson N. W. Fraley R. C. (2016a). Changing for the better? Longitudinal associations between volitional personality change and psychological well-being. Personality and Social Psychology Bulletin, 42(5), 603–615. 10.1177/0146167216637840 27016068

[bibr25-08902070231211957] Hudson N. W. Fraley R. C. (2016b). Do people’s desires to change their personality traits vary with age? An examination of trait change goals across adulthood. Social Psychological and Personality Science, 7(8), 847–856. 10.1177/1948550616657598

[bibr26-08902070231211957] Hudson N. W. Fraley R. C. Chopik W. J. Briley D. A. (2020). Change goals robustly predict trait growth: A mega-analysis of a dozen intensive longitudinal studies examining volitional change. Social Psychological and Personality Science, 11(6), 723–732. 10.1177/1948550619878423

[bibr27-08902070231211957] Hudson N. W. Roberts B. W. (2014). Goals to change personality traits: Concurrent links between personality traits, daily behavior, and goals to change oneself. Journal of Research in Personality, 53, 68–83. 10.1016/j.jrp.2014.08.008

[bibr28-08902070231211957] Hutteman R. Hennecke M. Orth U. Reitz A. K. Specht J. (2014). Developmental tasks as a framework to study personality development in adulthood and old age. European Journal of Personality, 28(3), 267–278. 10.1002/per.1959

[bibr29-08902070231211957] Kaiser T. Hennecke M. Luhmann M. (2020). The interplay of domain-and life satisfaction in predicting life events. PLoS One, 15(9), e0238992. 10.1371/journal.pone.0238992 32941489 PMC7498007

[bibr30-08902070231211957] Kroenke K. Spitzer R. L. Williams J. B. W. (2001). The PHQ-9: Validity of a brief depression severity measure. Journal of General Internal Medicine, 16(9), 606–613. 10.1046/j.1525-1497.2001.016009606.x 11556941 PMC1495268

[bibr31-08902070231211957] Leiner D. J. (2019). Too fast, too straight, too weird: Non-reactive indicators for meaningless data in internet surveys. Survey Research Methods, 13(3), 229–248. . 10.18148/srm/2019.v13i3.7403

[bibr32-08902070231211957] Lorah J. (2018). Effect size measures for multilevel models: Definition, interpretation, and TIMSS example. Large-Scale Assessments in Education, 6(1), 8. 10.1186/s40536-018-0061-2

[bibr33-08902070231211957] Lüdecke D. (2021). sjPlot: Data visualization for statistics in social science. https://CRAN.R-project.org/package=sjPlot

[bibr34-08902070231211957] Lüdecke D. Ben-Shachar M. S. Patil I. Makowski D. (2020). Extracting, computing and exploring the parameters of statistical models using R. Journal of Open Source Software, 5(53), 2445. 10.21105/joss.02445

[bibr35-08902070231211957] Luhmann M. Hennecke M. (2017). The motivational consequences of life satisfaction. Motivation Science, 3(1), 51–75. 10.1037/mot0000048

[bibr36-08902070231211957] Martínez-Martí M. L. Ruch W. (2014). Character strengths and well-being across the life span: Data from a representative sample of German-speaking adults living in Switzerland. Frontiers in Psychology, 5, 1253. 10.3389/fpsyg.2014.01253 25408678 PMC4219388

[bibr37-08902070231211957] McGrath R. E. (2019). The VIA assessment suite for adults: Development and initial evaluation. VIA Institute on Character.

[bibr38-08902070231211957] McGrath R. E. (2023). A summary of construct validity evidence for two measures of character strengths. Journal of Personality Assessment, 105(3), 302–313. 10.1080/00223891.2022.2120402 36121305

[bibr39-08902070231211957] McGrath R. E. Hall-Simmonds A. Goldberg L. R. (2020). Are measures of character and personality distinct? Evidence from observed-score and true-score analyses. Assessment, 27(1), 117–135. 10.1177/1073191117738047 29073771 PMC5878981

[bibr40-08902070231211957] McGrath R. E. Wallace N. (2021). Cross-validation of the VIA inventory of strengths-revised and its short forms. Journal of Personality Assessment, 103(1), 120–131. 10.1080/00223891.2019.1705465 31868546

[bibr41-08902070231211957] Miller T. J. (2022). Assessing the desire to change personality across methods. Journal of Personality Assessment, 104(4), 447–457. 10.1080/00223891.2021.1955695 34329561

[bibr42-08902070231211957] Miller T. J. Baranski E. N. Dunlop W. L. Ozer D. J. (2019). Striving for change: The prevalence and correlates of personality change goals. Journal of Research in Personality, 80, 10–16. 10.1016/j.jrp.2019.03.010

[bibr43-08902070231211957] Nakagawa S. Schielzeth H. (2013). A general and simple method for obtaining R2 from generalized linear mixed-effects models. Methods in Ecology and Evolution, 4(2), 133–142. 10.1111/j.2041-210x.2012.00261.x

[bibr44-08902070231211957] Peterson C. Seligman M. E. P. (2004). Character strengths and virtues: A handbook and classification. Oxford University Press.

[bibr45-08902070231211957] Pievsky M. A. McGrath R. E. (2022). National valuing of character strengths and indicators of national development: A pilot study. Applied Research in Quality of Life, 17(2), 703–721. 10.1007/s11482-021-09938-2

[bibr46-08902070231211957] Proyer R. T. Gander F. Wellenzohn S. Ruch W. (2015). Strengths-based positive psychology interventions: A randomized placebo-controlled online trial on long-term effects for a signature strengths- vs. a lesser strengths-intervention. Frontiers in Psychology, 6. 10.3389/fpsyg.2015.00456 PMC4406142 25954221

[bibr47-08902070231211957] Proyer R. T. Ruch W. Buschor C. (2013). Testing strengths-based interventions: A preliminary study on the effectiveness of a program targeting curiosity, gratitude, hope, humor, and zest for enhancing life satisfaction. Journal of Happiness Studies, 14(1), 275–292. 10.1007/s10902-012-9331-9

[bibr48-08902070231211957] Quintus M. Egloff B. Wrzus C. (2017). Predictors of volitional personality change in younger and older adults: Response surface analyses signify the complementary perspectives of the self and knowledgeable others. Journal of Research in Personality, 70, 214–228. 10.1016/j.jrp.2017.08.001

[bibr49-08902070231211957] R Core Team. (2022). R: A language and environment for statistical computing [manual]. https://www.R-project.org/

[bibr50-08902070231211957] Reisz Z. Boudreaux M. J. Ozer D. J. (2013). Personality traits and the prediction of personal goals. Personality and Individual Differences, 55(6), 699–704. 10.1016/j.paid.2013.05.023

[bibr51-08902070231211957] Revelle W. (2022). psych: Procedures for psychological, psychometric, and personality research [Manual]. https://CRAN.R-project.org/package=psych

[bibr52-08902070231211957] Roberts B. W. Walton K. E. Viechtbauer W. (2006). Patterns of mean-level change in personality traits across the life course: A meta-analysis of longitudinal studies. Psychological Bulletin, 132(1), 1–25. 10.1037/0033-2909.132.1.1 16435954

[bibr53-08902070231211957] Robinson O. C. Noftle E. E. Guo J. Asadi S. Zhang X. (2015). Goals and plans for Big Five personality trait change in young adults. Journal of Research in Personality, 59, 31–43. 10.1016/j.jrp.2015.08.002

[bibr54-08902070231211957] Ruch W. Niemiec R. M. McGrath R. E. Gander F. Proyer R. T. (2020). Character strengths-based interventions: Open questions and ideas for future research. The Journal of Positive Psychology, 15(5), 680–684. 10.1080/17439760.2020.1789700

[bibr55-08902070231211957] Ruch W. Proyer R. T. Harzer C. Park N. Peterson C. Seligman M. E. P. (2010). Values in action inventory of strengths (VIA-IS): Adaptation and validation of the German version and the development of a peer-rating form. Journal of Individual Differences, 31(3), 138–149. 10.1027/1614-0001/a000022

[bibr56-08902070231211957] Ruch W. Vylobkova V. Heintz S. (2023). Two of a kind or distant relatives? A multimethod investigation of the overlap between personality traits and character strengths. Journal of Individual Differences. 44(4), 263–270. 10.1027/1614-0001/a000400

[bibr57-08902070231211957] Scheinin I. Kalimeri M. Jagerroos V. Parkkinen J. Tikkanen E. Würtz P. Kangas A. (2023). ggforestplot: Forestplots of measures of effects and their confidence intervals. https://nightingalehealth.github.io/ggforestplot/index.html

[bibr58-08902070231211957] Stahlmann A. G. Ruch W. (2020). Scrutinizing the criteria for character strengths: Laypersons assert that every strength is positively morally valued, even in the absence of tangible outcomes. Frontiers in Psychology, 11, 591028. 10.3389/fpsyg.2020.591028 33101158 PMC7554639

[bibr59-08902070231211957] Stieger M. Eck M. Rüegger D. Kowatsch T. Flückiger C. Allemand M. (2020). Who wants to become more conscientious, more extraverted, or less neurotic with the help of a digital intervention? Journal of Research in Personality, 87, 103983. 10.1016/j.jrp.2020.103983

[bibr60-08902070231211957] Su R. Tay L. Diener E. (2014). The development and validation of the comprehensive inventory of thriving (CIT) and the brief inventory of thriving (BIT). Applied Psychology: Health and Well-Being, 6(3), 251–279. 10.1111/aphw.12027 24919454

[bibr61-08902070231211957] Sun J. Goodwin G. P. (2020). Do people want to be more moral? Psychological Science, 31(3), 243–257. 10.1177/0956797619893078 32045329

[bibr62-08902070231211957] Sun J. Wilt J. Meindl P. Watkins H. M. Goodwin G. P. (2023). How and why people want to be more moral. Journal of Personality. 10.1111/jopy.12812 36652292

[bibr63-08902070231211957] Thielmann I. de Vries R. E. (2021). Who wants to change and how? On the trait-specificity of personality change goals. Journal of Personality and Social Psychology, 121(5), 1112–1139. 10.1037/pspp0000304 33475400

[bibr64-08902070231211957] Wagner L. Gander F. Proyer R. T. Ruch W. (2020). Character strengths and PERMA: Investigating the relationships of character strengths with a multidimensional framework of well-being. Applied Research in Quality of Life, 15(2), 307–328. 10.1007/s11482-018-9695-z

[bibr65-08902070231211957] Wagner L. Pindeus L. Ruch W. (2021). Character strengths in the life domains of work, education, leisure, and relationships, and their associations with flourishing. Frontiers in Psychology, 12, 597534. 10.3389/fpsyg.2021.597534. 33967881 PMC8096931

[bibr66-08902070231211957] Wickham H. (2016). ggplot2: Elegant graphics for data analysis. Springer. https://ggplot2.tidyverse.org

[bibr67-08902070231211957] Wickham H. François R. Henry L. Müller K. (2023). dplyr: A grammar of data manipulation [Manual].https://CRAN.R-project.org/package=dplyr

